# Investigating Sequential and Simultaneous Changes in Trajectories of Cognitive Decline and Depressive Symptoms

**DOI:** 10.1093/geroni/igab046.285

**Published:** 2021-12-17

**Authors:** Matthias Klee, Anja Leist

**Affiliations:** 1 University of Luxembourg, Esch-sur-Alzette, Grevenmacher, Luxembourg; 2 University of Luxembourg, Esch-sur-Alzette, Diekirch, Luxembourg

## Abstract

Background. The role of depression as risk factor or early symptom of cognitive decline and dementia is still debated. Exploiting longitudinal trajectories of memory recall in a large European sample, we sought to better understand the nature of simultaneous versus sequential changes in depressive symptoms alongside memory recall at older ages. Method. A total of 4,865 respondents to the SHARE survey, mean age at t1 61.5 years (SD = 7.53), completed the EURO-D depression scale and a delayed recall task across six waves spanning ~13 years. We applied k-means clustering to distinguish trajectories of depressive symptoms and delayed recall. Clusters indicating depressive and recall trajectories were included in logistic regressions to assess likelihood of parallel versus sequential change, controlling for age, gender, employment status and education. Results. Analyses revealed six distinct trajectories each for depressive symptoms and delayed recall. Visual inspections indicated that only declining recall trajectories showed increases in depressive symptoms, occurring simultaneously rather than sequentially. Using grouped declining recall trajectories as outcome, the low-increasing depressive symptoms trajectory was associated with cognitive decline (OR = 1.52 [1.11, 2.06]), whereas the stable-high depressive symptoms trajectory was associated with cognitive decline in respondents aged 60-69 years (OR = 1.78 [1.01, 3.16]). Discussion. Distinguishing trajectories in depression and recall incorporates longitudinal information able to further elucidate relationships between depression and cognition. While the findings suggest depression as a co-morbidity, attention needs to be given to a comparatively small high-stable depressive symptoms trajectory group with elevated risk of cognitive decline in their 60s.

